# High-level inhibition of mitochondrial complexes III and IV is required to increase glutamate release from the nerve terminal

**DOI:** 10.1186/1750-1326-6-53

**Published:** 2011-07-26

**Authors:** Seán M Kilbride, Sonia A Gluchowska, Jayne E Telford, Catherine O'Sullivan, Gavin P Davey

**Affiliations:** 1 School of Biochemistry and Immunology & Trinity College Institute of Neuroscience, Trinity College Dublin, Dublin 2, Ireland

## Abstract

**Background:**

The activities of mitochondrial complex III (ubiquinol-cytochrome *c *reductase, EC 1.10.2.2) and complex IV (cytochrome *c *oxidase EC 1.9.3.1) are reduced by 30-70% in Huntington's disease and Alzheimer's disease, respectively, and are associated with excitotoxic cell death in these disorders. In this study, we investigated the control that complexes III and complex IV exert on glutamate release from the isolated nerve terminal.

**Results:**

Inhibition of complex III activity by 60-90% was necessary for a major increase in the rate of Ca^2+^-independent glutamate release to occur from isolated nerve terminals (synaptosomes) depolarized with 4-aminopyridine or KCl. Similarly, an 85-90% inhibition of complex IV activity was required before a major increase in the rate of Ca^2+^-independent glutamate release from depolarized synaptosomes was observed. Inhibition of complex III and IV activities by ~ 60% and above was required before rates of glutamate efflux from polarized synaptosomes were increased.

**Conclusions:**

These results suggest that nerve terminal mitochondria possess high reserves of complex III and IV activity and that high inhibition thresholds must be reached before excess glutamate is released from the nerve terminal. The implications of the results in the context of the relationship between electron transport chain enzyme deficiencies and excitotoxicity in neurodegenerative disorders are discussed.

## Background

Glutamate excitotoxicity is thought to occur in chronic neurodegenerative disorders such as Alzheimer's disease, Parkinson's disease, Huntington's disease and amyotrophic lateral sclerosis [1,2], and dysfunction of mitochondrial electrion transport chain complexes have been implicated in the pathogenesis of these diseases [3-5]. Reductions in complex II/III activity are specific to the brain areas affected by the pathogenesis of Huntington's disease [6-8]; decreased complex II/III activity in the caudate (by 29%) and putamen (by 67%) was found in post-mortem brain tissue, and complex IV activity was reduced in both regions by 30% and 62% respectively  [6,9]. Complex III deficiencies which occur as a result of rare mutations can result in the pathogenesis of encaphalpathic syndromes of various severity [10,11].

Widespread neurodegeneration throughout the brain has been shown to occur in Alzheimer's disease, and postmortem studies on the Alzheimer brain found that complex IV activity was reduced by 27% in the cerebral cortex, by 37% in the temporal cortex, and by 52% in the hippocampus [12,13]. Reductions in the activities of other mitochondrial enzymes, including complex III, have also been found [14,15]. A decrease in complex IV activity in the brain associated with aging is also thought to occur [16-18] and insufficient control over glutamate release due to mitochondrial complex III and IV deficiency are thought to contribute to neuronal cell death [8,19]. During conditions of severe energy stress, release of glutamate occurs primarily via reversal of plasma membrane glutamate transporters [20]. The depletion of intracellular ATP results in plama membrane depolarization and Ca^2+^-independent release of glutamate from the cytoplasmic pool. The resulting increase in extracellular glutamate concentration causes post-synaptic glutamate receptor overactivation, resulting in Ca^2+ ^overload and 'excitotoxic' cell death, Although most commonly associated with the pathogenesis of acute neurodegenerative disorders such as stroke and brain trauma, there is evidence suggesting that a similar 'slower' form of excitotoxicity contributes to chronic neurodegeneration [21].

Previous studies have examined the effects of total inhibition of complex IV activity using NaCN on glutamate release [22-25], which indicated that an increase in Ca^2+^-independent glutamate efflux from polarized synaptosomes occurs due to severe depletion of nerve terminal ATP content [24]. Under these conditions, insufficient ATP supply to the plasma membrane Na^+^/K^+ ^ATPase results in depolarization, and Ca^2+^-independent release of glutamate via reversal of glutamate transporters [26]. Complex I exerts a high level of flux control over oxidative phosphorylation in *in situ *synaptosomal mitochondria [27] and a 40% inhibition of complex I activity results in an increase in Ca^2+^-independent glutamate release from depolarized synaptosomes [28]. However, the effects of partial inhibition of complexes III and IV on glutamate release from nerve terminal preparations remain unknown.

Complexes III and IV have been shown to have high thresholds of inhibition of activity before major changes in oxygen consumption and ATP production occur in isolated brain mitochondria [29,30]. Comparison of such data obtained from experiments carried out on isolated nerve terminal mitochondria [31] with nonsynaptic mitochondria [32,33] indicate the threshold levels are higher in synaptosomal mitochondria for both complex III and complex IV respectively. This suggests that complexes III and IV have relatively low levels of control over oxidative phosphorylation in isolated synaptosomal mitochondria. Recently we demonstrated that both complex III and complex IV have lower control over oxygen consumption in *in situ *synaptosomal mitochondria than complex I [27]. To examine the control of complexes III and IV over glutamate release from nerve terminals, experiments using ranges of concentrations of the complex III inhibitors myxothiazol and antimycin A, which inhibit complex III activity upstream and downstream of the Q-cycle respectively [34], and a range of concentrations of the complex IV inhibitor KCN on glutamate release rates were carried out. Such data may be relevent to elucidating the role of excitotoxicity in the pathogenesis of neurodegenerative disorders.

## Results

### Complex III-related loss of nerve terminal control over glutamate release

**Figure 1:**
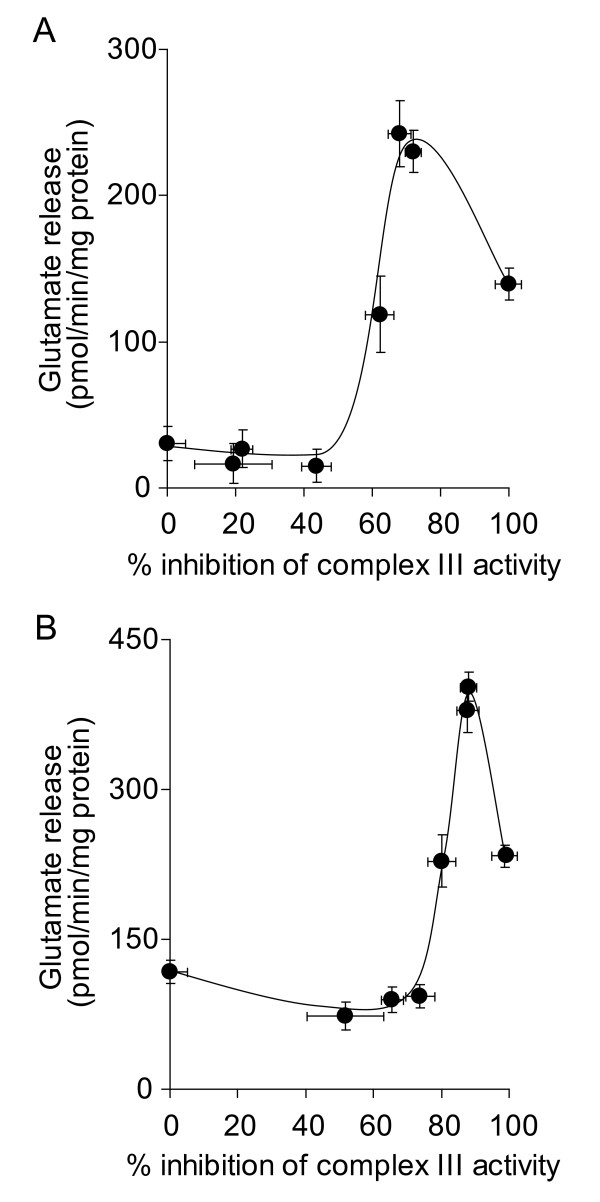
**High-level inhibition of complex III activity with myxothiazol is required to increase the rate of glutamate release from depolarized synaptosomes**. Synaptosomes (0.5 mg/ml) were preincubated at 37°C for 5 min before being depolarized with **A **1 mM 4-aminopyridine or **B **40 mM KCl. Rates of glutamate release at each concentration of myxothiazol were plotted against percent inhibition of complex III activity brought about by that concentration of myxothiazol. Freehand curves were drawn through the results. Points shown represent the mean ± SEM for experiments carried out in triplicate on at least 3 separate synaptosomal preparations.

**Figure 2:**
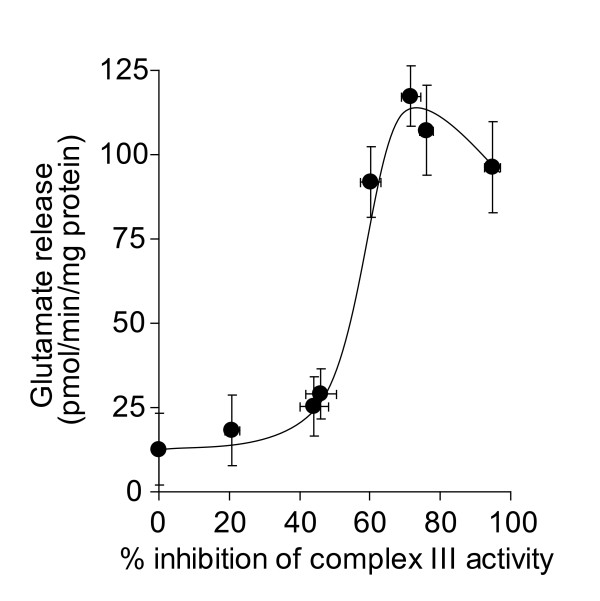
**High-level inhibition of complex III activity with myxothiazol is required to increase the rate of glutamate efflux from polarized synaptosomes**. Synaptosomes (0.5 mg/ml) were preincubated at 37°C for 5 min. Rates of glutamate release at each concentration of myxothiazol were plotted against percent inhibition of complex III activity brought about by that concentration of myxothiazol. Freehand curves were drawn through the results. Points shown represent the mean ± SEM for experiments carried out in triplicate on at least 3 separate synaptosomal preparations.

We have previously demonstrated that inhibition of complex III activity in synaptic mitochondria by up to 80% does not result in any major reductions in oxidative phosphorylation [33]. This suggests that a high threshold of inhibition of complex III activity must be exceeded before effects on mitochondrial oxygen consumption and ATP production occur. However, this threshold was found to be lower in *in situ *synaptosomal mitochondria [27]. A low threshold of inhibition was consistantly shown to exist for complex I [27,35], which correlated to a loss of synaptosomal control over Ca^2+^-independent glutamate release at low level inhibition of complex I activity [28,36]. In the present study, we examined the downstream effects of inhibition of complex III activity on synaptosomal control over Ca^2+^-independent glutamate release. We found that inhibition of complex III activity by > 60% increased glutamate release to almost 250 pmol/min/mg protein from synaptosomes depolarized with 4-aminopyridine (Figure 1A). However, low-level inhibition did not effect glutamate release, evidence that a complex III inhibition threshold must be exceeded before synaptosomal control over cytoplasmic glutamate retention is lost. Furthermore, inhibition by up to 80% did not affect KCl-induced Ca^2+^-independent glutamate release (Figure 1B), suggesting the occurrence of a similar threshold of inhibition to that found in isolated synaptic mitochondria. Inhibition of complex III activity by > 60% also increased glutamate efflux from synaptosomes in the absence of either depolarizing agent, although the peak rate of release was slower (~100 pmol/min/mg protein, Figure 2).

**Figure 3:**
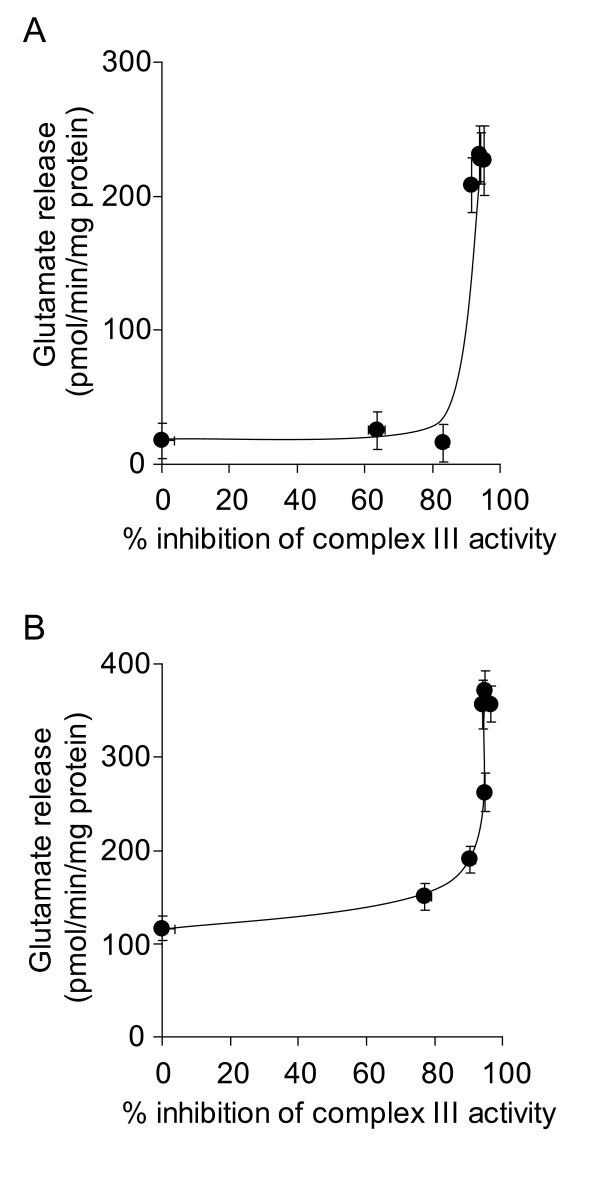
**High-level inhibition of complex III activity with antimycin A is required to increase the rate of glutamate release from depolarized synaptosomes**. Synaptosomes (0.5 mg/ml) were preincubated at 37°C for 5 min before being depolarized with **A **1 mM 4-aminopyridine or **B **40 mM KCl. Rates of glutamate release at each concentration of antimycin A were plotted against percent inhibition of complex III activity brought about by that concentration of antimycin A. Freehand curves were drawn through the results. Points shown represent the mean ± SEM for experiments carried out in triplicate on at least 3 separate synaptosomal preparations.

**Figure 4:**
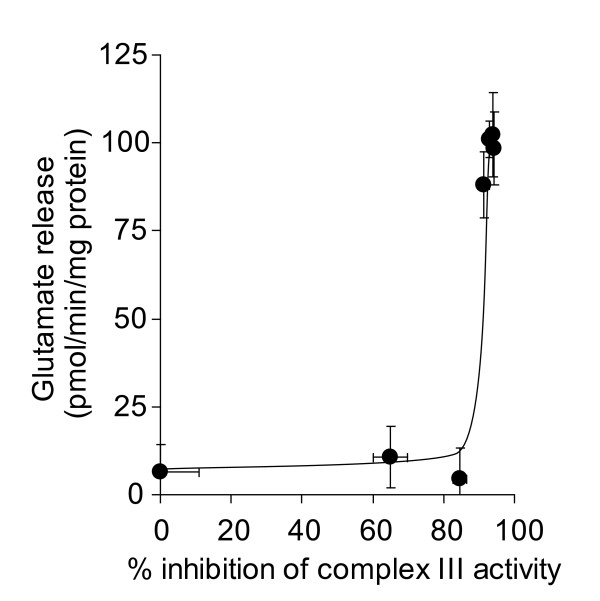
**High level inhibition of complex III activity with antimycin A is required to increase the rate of glutamate efflux from polarized synaptosomes**. Synaptosomes (0.5 mg/ml) were preincubated at 37°C for 5 min. Rates of glutamate release at each concentration of antimycin A were plotted against percent inhibition of complex III activity brought about by that concentration of antimycin A. Freehand curves were drawn through the results. Points shown represent the mean ± SEM for experiments carried out in triplicate on at least 3 separate synaptosomal preparations.

Inhibition of complex III activity at the Qi site with antimycin A also increased glutamate release in depolarized synaptosomes (Figures 3A &3B). However, inhibition by > 90% was required to elicit the increase, and a similar level of inhibition was required to increase glutamate efflux from polarized synaptosomes (Figure 4). This suggests that the inhibition thresholds that are required to be exceeded before glutamate release is effected may be heterogeneous within a single respiratory complex, depending on the site of inhibition.

### Complex IV-related loss of nerve terminal control over glutamate release

**Figure 5:**
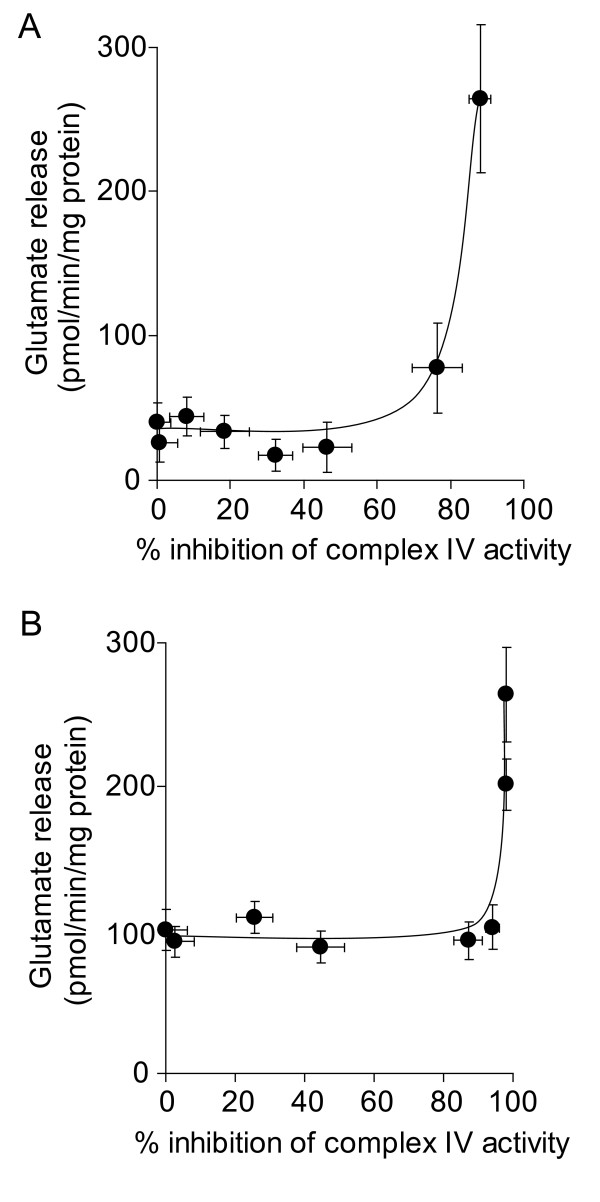
**High-level inhibition of complex IV activity with KCN is required to increase the rate of glutamate release from depolarized synaptosomes**. Synaptosomes (0.5 mg/ml) were preincubated at 37°C for 5 min before being depolarized with **A **1 mM 4-aminopyridine or **B **40 mM KCl. Rates of glutamate release at each concentration of KCN were plotted against percent inhibition of complex IV activity brought about by that concentration of KCN. Freehand curves were drawn through the results. Points shown represent the mean ± SEM for experiments carried out in triplicate on at least 3 separate synaptosomal preparations.

**Figure 6:**
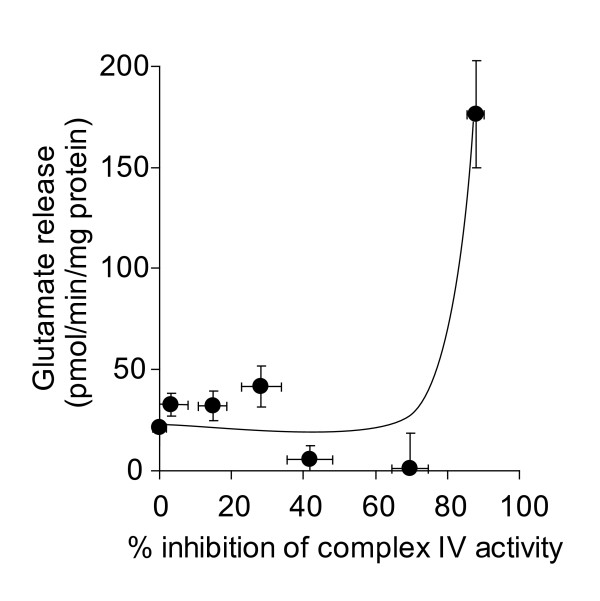
**High-level inhibition of complex IV activity with KCN is required to increase the rate of glutamate efflux from polarized synaptosomes**. Synaptosomes (0.5 mg/ml) were preincubated at 37°C for 5 min and rates of glutamate release at each concentration of KCN were plotted against percent inhibition of complex IV activity brought about by that concentration of KCN. Freehand curves were drawn through the results. Points shown represent the mean ± SEM for experiments carried out in triplicate on at least 3 separate synaptosomal preparations.

It has been established that high thresholds of inhibition of complex IV activity must to be exceeded before oxygen consumption is effected in nerve terminal mitochondria, evidenced in both isolated [31,33] and *in situ *[27] models. Total inhibition of complex IV activity using NaCN has been used as a model of anoxia, and has been shown to increase glutamate efflux from resting synaptosomes [22,23]. Here, we used a range of concentrations of KCN to establish the extent that complex IV activity controls glutamate release from depolarized and polarized synaptosomes. We found that 90% inhibition of complex IV activity was required to increase the rate of Ca^2+^-independent glutamate release from synaptosomes depolarized with 4-aminopyridine (Figure 5A). Overcoming a similarly high inhibition threshold was required to elicit an increase in glutamate release from synaptosomes depolarized with KCl (Figure 5B). In resting synaptosomes, in accordance with previous studies, we found that 1 mM KCN increased glutamate efflux, correlating to ~ 90% inhibition of complex IV activity (Figure 6). However, the rate was not affected by any lower concentration of KCN.

### ATP depletion caused by inhibition of complex III and IV

**Figure 7:**
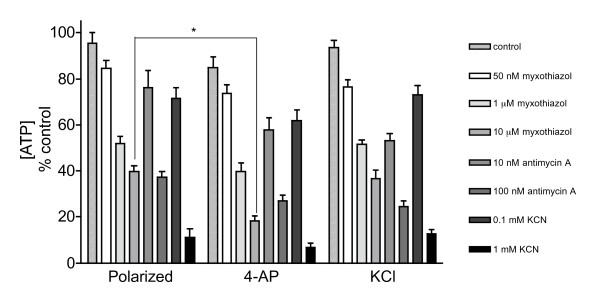
**Partial inhibition of complexes III and IV depletes ATP levels in synaptosomes**. Synaptosomes (0.5 mg/mL) were divided into groups and incubated at 37°C with 10 mM glucose and inhibitors as indicated. ATP from polarized synaptosomes was PCA extracted after 15 min incubation. In the depolarised groups 4-AP and KCl were added following 5 min incubation with inhibitors. Results are expressed as a percentage of the initial [ATP] at zero time (439.4 ± 42.6 pmol/mg protein) and represent the mean ± SEM for experiments carried out on three separate synaptosomal preparations. *p < 0.05.

In the absence of complex III or IV inhibitors, polarized and depolarized synaptosomes maintained their ATP levels close to control levels (Figure 7). Incubation with 50 nM myxothiazol reduced the ATP levels to approximately 80% of the control levels in all 3 groups. Myxothiazol concentrations of 1 and 10 μM lowered the ATP levels to 52 and 40% in polarized synaptosomes, respectively. However, depolarization with 4-aminopyridine potentiated the myxothiazol-induced ATP depletion to 40 and 18% with 1 and 10 μM myxothiazol, respectively. Addition of 100 nM antimycin A reduced ATP levels to 38% in polarized synaptosomes and to 28% in depolarized synaptosomes. Inhibition of complex IV activity by 0.1 and 1 mM KCN significantly reduced ATP levels to approximately 70% and 10%, respectively, in both polarized and depolarized synaptosomes. 

## Discussion

The results in this study indicate that high level inhibition of complex III and IV activities of *in situ *synaptosomal mitochondria are required to increase Ca^2+^-independent glutamate release rates from depolarized synaptosomes, as well as the Ca^2+^-independent glutamate efflux rate from resting synaptsomes. Inhibition of complex III activity by more than 50% with myxothiazol was required to increase glutamate release from depolarized and polarized synaptosomes. Complex III activity could be inhibited by up to 90% with antimycin A before KCl- or 4-aminopyridine-induced release or glutamate efflux was affected. A similarly high threshold of inhibition was found when complex IV was inhibited with KCN. Previously we have shown that inhibition of complex I activity by approximately 40% increases the rate of Ca^2+^-independent glutamate release from depolarized synaptosomes [28]. This suggests that complex I has greater control over the release cytosolic pool of glutamate in the depolarized nerve terminal model than complex III or complex IV. However, we also showed that in polarized synaptosomes, glutamate efflux was not affected by any level of inhibition of complex I activity [28], whereas in the present study it was demonstrated that inhibition of complex III or complex IV caused an increase in glutamate efflux at the highest levels of inhibition.

Using a range of myxothiazol concentrations, inhibition of complex III activity by up to 80% did not result in major changes in oxidative phosphorylation, while inhibition by 90% completely abolished the oxygen consumption and ATP production in mitochondria isolated from synaptosomes [33]. However, the threshold level of inhibition of complex III activity on oxygen consumption in *in situ *synaptosomal mitochondria was found to be lower than that reported in isolated synaptic mitochondria [27]. In the *in situ *synaptosomal mitochondrial model, inhibition of complex III activity by 70% with myxothiazol was found to almost completely abolish the oxygen consumption rate [27]. At this level of inhibition mitochondrial ATP synthesis is also minimal (as oxygen consumption is tightly coupled to ATP synthesis in nerve terminals [37]). In the present study we found that ATP levels in polarized synaptosomes were decreased by 60% by 10 μM myxothiazol. Under resting conditions glycolysis is thought to account for the remaining ATP synthesis [37]. Glutamate efflux from 'polarized' synaptosomes was found to occur at a maximum of 70% inhibition of complex III activity and Ca2+-independent glutamate release from synaptosomes depolarized with 4-aminopyridine over the same range of inhibition of complex III activity with myxothiazol (between 50 - 70%) was 2.5-fold higher than from polarized synaptosomes. This increase in the rate of glutamate release correlated with an extra 20% reduction in ATP levels induced by 4-aminopyridine in combination with myxothiazol-related inhibition of complex III activity. This suggests that the control of complex III over oxidative phosphorylation in synaptosomes is similar to the control of complex III over glutamate release rates from synaptosomes.

Interestingly, glutamate release returned towards control levels as inhibition of complex III neared 100%. This is similar to the effect observed on Ca^2+^-independent glutamate release from depolarized synaptosomes following inhibition of complex I activity [28]. A short-term plasma membrane potential hyperpolarization response by neurons to extreme energy stress has been previously demonstrated [39]. This phenomenon may be related to the presence of ATP-sensitive K^+ ^channels on the plasma membrane [38], which open during periods of severe ATP depletion (correlating to an approximately 90% reduction in ATP levels in our experiments), and are thought to allow short term maintainance of the K^+ ^gradient across the plasma membrane when the Na^+^/K^+ ^ATPase activity is compromised [39].

The highest rates of Ca^2+^-independent glutamate release brought about by complex III inhibition with antimycin A were similar to those with myxothiazol. However, 80% inhibition of complex III activity with antimycin A did not increase Ca^2+^-independent glutamate release rates from synaptosomes depolarized with 4-aminopyridine, while maximum 4-aminopyridine-induced glutamate release rates were recorded at less than 80% inhibition of complex III activity with myxothiazol. Similarly, 80% inhibition of complex III activity with antimycin A did not increase glutamate efflux rates from 'polarized' synaptosomes while maximum glutamate efflux rates were recorded at less than 80% inhibition of complex III activity with myxothiazol. This suggests that complex III of *in situ *nerve terminal mitochondria may have a lower energy threshold when inhibited upstream of the Q-cycle with myxothiazol than when inhibited downstream with antimycin A, which results in increased rates of glutamate release at a lower level of inhibition.

Inhibition of complex IV activity by > 90% with KCN also increased Ca^2+^-independent glutamate release rates from synaptosomes depolarized with 4-aminopyridine or KCl, and increased glutamate efflux from 'polarized' synaptosomes. A similar increase in glutamate efflux has previously been reported in rat brain synaptosomes when 2 mM NaCN was added, and was related to a fall in ATP/ADP ratio [24]. Inhibition of complex IV activity by up to 70% with KCN in isolated synaptosomal mitochondria has previously been shown to decrease ATP production by less than 10%, with total inhibition of ATP production at 90% inhibition of complex IV activity [33]. The similar threshold effect found in *in situ *synaptosomal mitochondria [27] might explain the effects of complex IV inhibition on Ca^2+^-independent glutamate release from depolarized synaptosomes.

## Conclusions

Reduced complex II/III activity may play a role in the excitotoxic mechanism thought to occur during the pathogenesis of Huntington's disease [6-8], and reduced complex IV activity has been consistantly observed in post mortem Alzheimer's disease brain samples [14]. Neurodegeneration has been proposed to progress from the nerve terminal to the neuronal soma in these chronic neurdegenerative disorders [40]. Indeed, ETC enzyme activities in the brain have been shown to be reduced as part of the aging process [41,42]. However, the model of the effects of reduced activities of complex III and complex IV nerve terminal glutamate release in the present study indicate that these enzymes must be inhibited by at least 50% (for complex III) or 90% (for complex IV) before major increases in glutamate release rates are observed. Such large reductions in activity are usually related to mutations in either the mitochondrial or nuclear DNA encoding components of the complexes, and usually result in severe metabolic disorders which are often fatal [10,11]. However, our model may support a role for excessive nerve terminal glutamate release during pathogenesis of encephalopathies caused by complex III and IV deficiencies, in which seizures are commonly reported.

A lower level of inhibition of complex I activity is required to reduce nerve terminal oxygen consumption [27] and increase Ca^2+^-independent glutamate release rates from depolarized nerve terminals than complex III or IV [28]. Nonetheless, inhibition of complex I activity by up to 100% did not affect glutamate efflux from resting synaptosomes [28], while inhibition of complexes III and IV increased the efflux rate, although a high level of inhibition was required. This indicates that both complex III and complex IV exert greater control over glutamate release from resting nerve terminals than complex I. However, given that the complex I threshold for Ca^2+^-independent glutamate release in depolarized synaptosomes isolated from aged rats is even lower than that from young rats [36] coupled with the findings that complex I activity is decreased in mitochondria isolated from Parkinson's disease post mortem samples [43-45], the evidence is mounting that of the ETC enzymes that are found to be reduced in the most common chronic neurodegenerative disorders, complex I is the most important.

## Materials and methods

### Materials

Chemicals were supplied by Sigma Chemical Co., Poole, Dorset, UK or BDH, Dagenham, Essex, UK. Female Wistar rats (200-250 g) were supplied by the Bioresources Unit, Biochemistry Department, Trinity College, Dublin.

### Synaptosomal preparation

Rats were killed by cervical dislocation and synaptosomes were prepared using a discontinuous ficoll gradient (7.5% w/v and 10% w/v), acording to the method of Lai and Clark [46]. Synaptosomes (1 mg) were resuspended in TES buffer (250 mM sucrose/5 mM TES, pH 7.4) and following centrifugation at 15000 g for 5 min were stored as 1 mg pellets on ice for use within 2 hours of preparation. All experiments were carried out on at least 3 separate synaptosomal preparations to ensure reproducibility of results.

### Glutamate release

Glutamate release was measured on a SpectraMAX GeminiXS (Molecular Devices, CA) well plate reader using a continuous flourimetric assay modified from that described by Nicholls *et al. *[47]. Synaptosomal pellets were resuspended in 1 ml of incubation medium (3 mM KCl, 140 mM NaCl, 25 mM Tris-HCl, 10 mM glucose, 2 mM MgCl_2_, pH 7.4). 100 μl of incubation medium containing 2 mM NADP^+^, 6.32 U L-glutamic dehydrogenase, (and 1.4 mM CaCl_2 _where appropriate) was distributed into each of the 96 wells. 2 μl of each respective mitochondrial electron transport chain enzyme inhibitor (final concentrations as follows: myxothiazol, 10 nM - 10 μM; antimycin A, 1 nM - 1 μM; or KCN, 1 μM - 1 mM;) was added followed by 100 μl of resuspended synaptosomes (final concentration 0.5 mg/ml) and each experimental condition was carried out in triplicate on each plate. Synaptosomes were depolarized after 5 minutes and rate of increase in NADPH fluorescence at λ = 460 nm emission (340 nm excitation) was recorded over a 20 minute time period at a 32 second interval following depolarization. Linear rates were fitted to the traces by the SoftMax Pro program, which accompanies the instrument, and these rates were calibrated using a standard curve. Enzyme lag [47] was accounted for when converting rates to nmol/min/mg protein.

### Complex III activity assay

Complex III activity was measured using a SpectraMAX 340PC well-plate spectrophotometer. The titration of complex III activity with myxothiazol (10 nM - 10 μM) or antimycin A (1 nM - 1 μM) was carried out identically to the glutamate release experiments in which synaptosomes (0.5 mg/ml) were incubated with inhibitor a final volume of 202 μl. Samples were frozen at -80°C and subsequently underwent 3 rapid freeze-thaw cycles. Complex III activity was determined by following the reduction of cytochrome *c *at absorbance λ = 550 nm. Decylubiquinol was used as the electron donor and was prepared by reducing decylubiquinone with sodium borohydride and extraction under nitrogen in cylohexane/diehyl ether. Wells were prepared by addition of medium containing 100 mM potassium phosphate, 0.3 mM potassium-EDTA, (pH 7.4), 1 mM KCN and 100 μM cytochrome *c *with a final volume of 200 μl. The reaction was initiated by the addition of sample (10 μg) and results were expressed as a percentage of first order decay rate constants (k).

### Complex IV activity assay

Complex IV activity was measured using an Agilent ChemStation 8453 spectrophotometer. The titration of complex IV activity with KCN was also carried out identically to the glutamate release experiments, where synaptosomes (0.5 mg/ml) were incubated in a final volume of 202 μl with inhibitor. After freezing at -80°C and undergoing 3 rapid freeze-thaw cycles, the activity of complex IV was determined using the method of Wharton and Tzagoloff [48]: The oxidation of cytochrome *c *at absorbance λ = 550 nm was followed. Reduced cytochrome *c *was prepared by the addition a few crystals of ascorbic acid to oxidised cytochrome *c *(25 mg/2.5 ml in H_2_O). Excess ascorbic acid was removed by passing the cytochrome *c *sample through a PD_10 _gel filtration column, which had been pre-rinsed with 50 ml 1:10 (v/v) dilution of potassium phosphate buffer (100 mM), pH 7.0. The assay cuvettes contained 50 μM reduced cytochrome *c *and 100 μl buffer with a final volume of 1 ml made up with H_2_O. The reaction was initiated by the addition of 50 μg synaptosomal samples. The results were expressed as a percentage of first order decay rate constants (k).

### ATP determinations

ATP levels were determined using a luciferase coupled assay [28]. Synaptosomes (1 mg/mL) were pre-incubated at 37°C for 5 min in incubation medium containing 10 mM glucose. After 5 min 100 μL synaptosomes were added to 100 μL of incubation medium with 2 μL myxothiazol, antimycin or KCN added, where appropriate. One group was prepared for ATP determination immediately at this point, by adding 10 μL 6.5 M perchloric acid and, after centrifugation, neutralizing 150 μL of supernatant with 375 μL 1 M K_2_HPO_4_. Another group was depolarized after 5 min (with 1 mM 4-aminopyridine). [ATP] is expressed as a percentage of the control group that contained no complex III or IV inhibitors. These groups were perchloric acid extracted in the same way 10 min after depolarization and all samples were then stored at -80°C. 10 μL of extract was added to 100 μL reconstituted luciferin/luciferase ENLITEN^® ^reagent and read immediately at 560 nm. All samples were assayed within 1 week of preparation.

### Statistical analysis

Results presented are mean ± SEM values. Statistical analysis of the results were determined by doing a one-way ANOVA followed by a Newman-Keuls post-hoc test. Values of p < 0.05 were taken to be significant.

## Competing interests

The authors declare that they have no competing interests.

## Authors' contributions

SMK, SAG, JET and COS did all experimental work. SMK wrote the manuscript. GPD conceived of the study, and participated in its design and coordination and co-wrote the manuscript. All authors read and approved the final manuscript.
